# Supplementary material for: Exploring the experiences of cancer patients: What drives them to seek treatment outside their residential area and what are the experiences resulting from that decision? A qualitative study
Source: PLoS One. 2025 Mar 10;20(3):e0319650. doi: 10.1371/journal.pone.0319650 (PMC11892868; doi:10.1371/journal.pone.0319650)
Supplement: Attachment — Submitted filename: Review comments response_20240923.docx [file pone.0319650.s001.docx]

**Response to reviewers**

We would like to express our sincere gratitude for your valuable feedback and constructive comments on our manuscript. We have revised our manuscript in accordance with your suggestions. In the manuscript body, our revisions are in red font to make them easy for you to locate. We look forward to your reply.

**[Reviewer 1]**

Major Concerns
It is not clear exactly what this study is trying to achieve. The study's objectives are unclear due to inconsistent articulation of the study aim across the abstract, introduction, and discussion sections. This discrepancy makes it challenging to fully understand the study's intended goals.

Response: Thank you for your valuable feedback. This study conducted an in-depth exploration of the experiences of Korean cancer patients who received treatment at medical institutions outside their region. In the introduction of this study, the necessity of the research objective is demonstrated through four main points. First, considering the burden of cancer, it is essential to establish a robust cancer care infrastructure. Second, it is important to develop a regionally tailored cancer care system. Third, there is a need to explore the experiences of cancer patients who seek treatment outside of their residential areas. Lastly, due to the lack of existing research on this topic, this study aims to explore the experiences of cancer patients receiving treatment in different regions using qualitative research methodologies. In the discussion section, we highlighted the need for expanding high-quality cancer care system in each region, the necessity of physician-level quality assessments and public disclosure, and the importance of strengthening the coordination role of primary care in post-cancer management. Based on your feedback, we have reviewed the content and modified the background and discussion in the abstract to better align with the research objectives stated in the introduction and discussion sections of the manuscript (lines 54-61, 76-80).

The rationale for the study needs to be much better refined. The authors emphasise the need to better understand why patients seek cancer outside their areas of residency. However, as the authors previously highlight in the introduction, the centralization of cancer care in urban areas is clearly a primary factor driving this behaviour. In other words, patients often have no choice but to travel, or as alluded to, there are clear inequalities in healthcare access that perpetuate seeking care elsewhere. It might be more beneficial to rephrase the rationale of the study to reflect the importance of investigating the needs and experiences of cancer patients living in areas where there is limited or poor access to appropriate cancer care.

Response: Thank you for your valuable feedback. We have revised the background section in the abstract, and also in the main text, we have emphasized the importance of policies aimed at narrowing the socioeconomic disparities that can clearly arise from the centralization of cancer care by adding further references to support this need (lines 102, reference 16-19).

The study population consists of a sample of female cancer patients aged 30 to 60 with either stage 1 or 2 cancer. This sample is likely not representative of the broader cancer population in the area of Ulsan Metropolitan City. Females in this age range may have unique needs different from those of other genders, age groups, and more advanced cancers. This represents a major concern and significant limitation of the study. Additionally, the recruitment window was open for only 10 days (August 31st – September 9th). Why was this? Extending the recruitment period could have resulted in a more diverse and representative sample.

Response: Thank you for your valuable feedback. I understand your concerns, and I appreciate your thoughtful input. Qualitative research is not intended to provide representativeness but rather to capture and present the experiences of participants as they are. As you mentioned, some participants, particularly those in their 30s, faced unique challenges related to their life stage (e.g., raising children). While the categorization highlights the main common experiences of participants, specific experiences that were particularly significant to certain individuals were written separately. Nonetheless, to avoid any overinterpretation of the findings, I ensured that the study’s limitations are clearly stated (lines 516-520). Regarding the recruitment period, I kindly ask for your understanding that the recruitment was conducted over 10 days due to the overall research timeline.

**Abstract**

The conclusion of the abstract could be stronger. The study's findings indicate that cancer patients often travel to receive care elsewhere due to various reasons. A more striking and definitive conclusion should emphasise the importance of addressing potential barriers that discourage patients from seeking local treatment.

Response: Incorporating your feedback, we have revised the conclusion of the abstract to emphasize that overcoming various obstacles is essential for establishing a comprehensive regional cancer care system (lines 76-80).

**Introduction**

Line 85 ‘succumbing to it’ – what does this mean? Dying because of cancer? This needs to be better clarified.

Response: Thank you for your detailed feedback. ‘Succumbing’ has been modified to ‘dying’ (lines 85-86).

**Methods**

The study included a sample size of 7 participants. Whilst it is acknowledged that in qualitative research a sample size of 7 can be considered adequate, it’d like to see more of a rationale for this sample size in context of the research question, the complexity of the problem under investigation, and the method of analysis.

Response: We made efforts to ensure a transparent recruitment process for participants. Through the recruitment announcement, a total of 16 individuals expressed their willingness to participate. To ensure maximum variation sampling, the research team reviewed the residence, cancer stage, gender, age, and type of cancer of the 16 applicants and selected 11. Of these 11 selected participants, 4 were excluded due to refusal to consent, poor health status, or non-response, leaving a final total of 7 participants. Additionally, during the data collection process, the research team confirmed theoretical saturation regarding the participants’ experiences, and thus, no further participants were recruited.

It is crucial to contextualize the setting in which this study is conducted. The core aim is to understand why patients choose to travel elsewhere for cancer care and treatment. Therefore, it is important to provide a comprehensive understanding of the area, including details about the population, hospital infrastructure, and the distance to other cancer centres. This context will greatly assist readers in understanding the study's background and significance.

Response: Thank you for your detailed feedback. In addition to the sociodemographic information of the participants, we have included the number of general hospitals in their residential areas and the public transportation time from the center of their residence to the regional cancer center to aid the reader’s understanding (line 210, Table 1). In the method section, we also added a footnote providing details on the healthcare status of Ulsan Metropolitan City. Ulsan is administratively divided into four districts (Gu) and one county (Gun), with a total population of approximately 1.1 million as of July 2024. The city has one tertiary hospital, eight general hospitals, and one public healthcare facility. The regional cancer center is located within the tertiary hospital (line 140, footnote 1).

One of the inclusion criteria was "having experience of hospitalization for cancer treatment in a medical institution outside their residential area." This criterion is somewhat vague; it would be beneficial to clarify what is meant by "outside their residential area." Providing specific details about the distance or geographical boundaries will help readers understand the extent to which patients are traveling for cancer treatment.

Response: Thank you for your considerate feedback. ‘Outside of the residential area’ refers to locations other than the participant’s place of residence (on a city-level basis). A more detailed explanation of the participant selection criteria has been provided (line 141). Additionally, we added the following description of Ulsan in Footnote 2. South Korea is composed of 17 administrative regions, and Ulsan is one of them. These selection criteria refer to individuals who accessed medical institutions in the other 16 regions outside of Ulsan for the purpose of cancer treatment.

**Results**

The results presented are interesting and offer unique insights into why cancer patients choose to travel outside their area of residence for care. Most findings align with the overarching aim of understanding the reasons behind this travel. However, some results focus more on general experiences and facilitating factors during the patients' journeys. The study's aim should better reflect this broader scope—emphasizing not only why patients travel for care but also their overall experiences and the factors that facilitate their decisions.

Response: Thank you for your valuable feedback. In the process of comprehensively exploring the experiences of cancer patients receiving treatment in different regions, we were able to identify a range of experiences, including general experiences and facilitating factors. Upon review, I noticed that while the research objective presented at the end of the introduction addresses the points you mentioned, the objective described in the discussion section was somewhat lacking. I have since revised the research objective in the discussion section to ensure consistency and clarity (lines 436-441).

**Discussion**

Overall a good discussion section is presented. My only suggestions for this sections are as follows. 1) the aim needs to be consistent with what is stated in the abstract and introduction, 2) I'd like to see a better acknowledgement of the limitations of the study i.e. the lack of a diverse sample, limited recruitment window etc. and 3) I'd also like to see a brief discussion on how future research can begin to address these issues and start to develop and improve localised cancer services.

Response: Thank you for your kind suggestions to improve this research.

1. The aim in the discussion section have been revised to be consistent with those presented in the abstract and introduction (lines 436-441).
2. The limitations have been described in more detail (lines 516-520).
3. We have already proposed various suggestions in the manuscript to encourage localized cancer care. Specifically, we suggested expanding medical institutions capable of providing high-quality cancer treatment within each region, conducting individual physician-level quality assessments to gauge medical expertise and making the results publicly available, strengthening the coordinating role of primary care institutions in cancer care, and enhancing post-discharge cancer management systems for cancer patients.

**[Reviewer 2]**

First, the title of the study needs to match the aims of the current study.

Response: Thank you for your kind feedback. I have revised the research title to align with the study’s objective of capturing both the reasons why cancer patients seek care at medical institutions outside their residential area and their overall experiences resulting from that decision (lines 1-3).

The objectives stated in the abstract must match those stated in the introduction.

Response: I have revised the objectives stated in the abstract to align them with those specified in the introduction (lines 54-61).

Furthermore, the current study's introduction was excellent, but it needed to be more focused to make it shorter and more direct to the point.

Response: In the introduction of this study, the necessity of the research objective is demonstrated through four main points. First, considering the burden of cancer, it is essential to establish a robust cancer care infrastructure. Second, it is important to develop a regionally tailored cancer care system. Third, there is a need to explore the experiences of cancer patients who seek treatment outside of their residential areas. Lastly, due to the lack of existing research on this topic, this study aims to explore the experiences of cancer patients receiving treatment in different regions using qualitative research methodologies. We have condensed some sections of the introduction to convey the key points more directly (lines 85-86).

**Methodology**

The abstract should be comprehensive and not solely focused on the participants.

Response: I have comprehensively revised the background and conclusion sections of the abstract. This revision aims to help readers better understand the context of the study and the policy recommendations derived from the research findings (lines 54-61, 76-80).

Regarding the methodology, we need to provide more specific details about the first two categories. There is a shortage with the questions used; there must be a category regarding the economic impact, and the causes of travelling to other parts of the country to receive treatment need to be more clearly defined and deepened.

Response: Thank you for your kind feedback. This study utilized a semi-structured questionnaire. Although only three questions were officially used (lines 169-177), additional experiences were explored based on the flow of participants’ narratives. We already included information related to the economic impact in subcategory “2-2. Challenges of traveling for long-distance treatment” of the results section. Participants reported economic difficulties to a similar extent as physical and psychological challenges, so a separate category for economic impact was not presented. However, we have made efforts to more clearly define the reasons for traveling to other regions for treatment (lines 255-256, 278-280).

About discussion Please remove the first section; it seems like part of the introduction and methodology. The same is true about the coincidence.

Response: We have revised the first section of the discussion extensively based on your feedback. As a result, only the objectives of the study and the results presented in the figures remain (lines 436-441).
