# Supplementary material for: Exploring the experiences of cancer patients: What drives them to seek treatment outside their residential area and what are the experiences resulting from that decision? A qualitative study
Source: PLoS One. 2025 Mar 10;20(3):e0319650. doi: 10.1371/journal.pone.0319650 (PMC11892868; doi:10.1371/journal.pone.0319650)
Supplement: Attachment — Submitted filename: Review comments response_20241229.docx [file pone.0319650.s002.docx]

**Response to reviewers**

We would like to express our sincere gratitude for your valuable feedback and constructive comments on our manuscript. We have revised our manuscript in accordance with your suggestions. In the manuscript body, our revisions are in red font to make them easy for you to locate. We look forward to your reply.

**[Reviewer 1]**

1. Please attempt to better align the aims across the Abstract, Introduction, and Discussion. I recognise that you have already attempted this, however, they do not fully align. I'd recommend defining one clear aim and repeating it verbatim. The slight variations make it a confusing.

Abstract aim: In this study, we explored the treatment experiences of cancer patients who used medical institution outside their regions, aiming to identify issues within the local cancer care system and propose policy improvements.

Introduction aim: this study aims to conduct qualitative research involving cancer patients to thoroughly investigate their experiences with cancer care at hospitals in different regions, covering the entire care continuum from diagnosis and treatment to management.

Discussion aim: This study conducted a qualitative investigation on cancer patients in Korea to explore their overall experiences with cancer treatment, as well as their experiences receiving treatment at medical institutions located outside their residential area.
**Response: Thank you for your valuable feedback. Based on your suggestion, we have revised the aims in the abstract, introduction, and discussion to ensure consistency throughout the manuscript (lines 58-61, 119-122, 436-438).**

2. I fully understand that qualitative research primarily focuses on capturing experiences rather than aiming for objective representation. However, if your sample population is not representative of the broader cancer population, it would be inappropriate to make generalisations about the wider cancer population.

I would like to see a more thoughtful acknowledgment of this in the limitation section. For example 'The findings of this study should be interpreted with caution when generalising to broader cancer populations. The sample consists of females aged 30 to 60, primarily with early-stage breast, thyroid, and parotid cancers. The authors acknowledge the exclusion of other wider cancer populations as a major limitation e.g. those who are male, are older (aged 60 and above), have later-stage or advanced cancer, and have different cancer types. Future studies are strongly encouraged to employ purposive sampling techniques to ensure the study population is more representative of the broader cancer population.'

I would strongly encourage referring to and citing the following article:

Benoot C, Hannes K, Bilsen J. The use of purposeful sampling in a qualitative evidence synthesis: A worked example on sexual adjustment to a cancer trajectory. BMC Med Res Methodol. 2016 Feb 18;16:21. doi: 10.1186/s12874-016-0114-6. PMID: 26891718; PMCID: PMC4757966.

**Response: Thank you for your valuable feedback. We have revised the description of this study's limitations to provide greater clarity. Additionally, using the recommended reference, we emphasized the need for purposive sampling in future studies to address the limitations identified in this research (lines 515-528).**

**[Reviewer 2]**

Aims in the Introduction: I recommend revisiting and rewriting the aims of your study as presented in the introduction. The current version could be more closely aligned with the study's title, results, and discussion. The aims you included in the abstract appear to be more consistent and reliable in relation to the overall study. Please consider revising the introduction to reflect a similar approach.

**Response: Thank you for your valuable feedback. Based on your suggestion, we have revised the aims in the abstract, introduction, and discussion to ensure consistency throughout the manuscript (lines 58-61, 119-122, 436-438).**

Conclusion: It is important to concisely summarize the main findings of your study in the conclusion. In addition, I suggest incorporating recommendations based on your findings, without expanding on what the results may potentially lead to. The focus should remain on the study's key outcomes and their direct implications.

**Response: Thank you for your valuable feedback. In the conclusion, we have concisely summarized the key findings of the study. Additionally, we have revised the content to focus on the specific recommendations derived from the study's results (lines 533-538).**
